# Childhood conduct problems and parent–child talk during social and nonsocial play contexts: a naturalistic home-based experiment

**DOI:** 10.1038/s41598-024-51656-w

**Published:** 2024-01-10

**Authors:** Sydney Sun, Rista C. Plate, Callie Jones, Yuheiry Rodriguez, Chloe Katz, Melissa Murin, Jules Pearson, Julia Parish-Morris, Rebecca Waller

**Affiliations:** 1 Department of Psychology, University of Pennsylvania, Levin Building, 425 S. University Ave., Philadelphia, PA 19104 USA; 2 Center for Autism Research, Children’s Hospital of Philadelphia, Philadelphia, PA USA; 3 Department of Child and Adolescent Psychiatry and Behavioral Sciences, Children’s Hospital of Philadelphia, Philadelphia, PA USA; 4 Department of Psychiatry, Perelman School of Medicine, University of Pennsylvania, Philadelphia, PA USA

**Keywords:** Human behaviour, Psychology

## Abstract

Parent–child interactions are a critical pathway to emotion socialization, with disruption to these processes associated with risk for childhood behavior problems. Using computational linguistics methods, we tested whether (1) play context influenced parent–child socioemotional language, and (2) child conduct problems or callous-unemotional traits were associated with patterns of socioemotional or nonsocial language across contexts. Seventy-nine parent–child dyads (children, 5–6 years old) played a socioemotional skills (“social context”) or math (“nonsocial context”) game at home. We transcribed and analyzed game play, which had been audio recorded by participants. The social context elicited more socioemotional and cognitive words, while the nonsocial context elicited more mathematical words. The use of socioemotional language by parents and children was more strongly correlated in the social context, but context did not moderate the degree of correlation in cognitive or mathematical word use between parents and children. Children with more conduct problems used fewer socioemotional words in the social context, while children with higher callous-unemotional traits used fewer cognitive words in both contexts. We highlight the role of context in supporting socioemotionally rich parent–child language interactions and provide preliminary evidence for the existence of linguistic markers of child behavior problems. Our results also inform naturalistic assessments of parent–child interactions and home-based interventions for parents and children facing socioemotional or behavioral challenges.

## Introduction

Emotional language is fundamental to successful social interactions and plays a central role in human perceptions, actions, and experiences^1^. On average, children demonstrate an understanding of specific emotion words (e.g., happy, sad) by age 2^2^ and produce complex emotion words (e.g., surprise, disgust) by age 4^3^. Children’s emotional language comprehension and expression increase dramatically across development, roughly doubling every second year until adolescence^3^. This development occurs synergistically with broader childhood socioemotional development^4–6^ and precedes the emergence of key social skills, such as the recognition of facial signals of emotion and emotion regulation^5,7^. Children who are less proficient at verbalizing social or emotional concepts show difficulties with social functioning more broadly^8^. Children’s socioemotional language could thus serve as an objective marker of risk for future difficulties in social functioning or behavior.

In particular, conduct problems (e.g., aggression, bullying, and deceitfulness) represent one of the most common reasons for childhood referrals to mental health services^9^. Moreover, children with early conduct problems are at elevated risk for chronic antisocial behavior and other forms of psychopathology across the lifespan^10^. Childhood conduct problems are linked to general language difficulties, including in language mechanics (e.g., trouble with grammar), emotional expression (e.g., restricted ability to verbalize feelings), and vocabulary (e.g., limited range of word use)^11–14^. In concert, children with conduct problems have difficulties responding appropriately to and managing complex social situations^15,16^, expressing empathy^17^, and developing social relationships^18^. Restricted empathic displays and low social bonding are particularly pronounced among a subgroup of children with conduct problems and co-occurring callous-unemotional traits^19,20^, who show specific difficulties recognizing distress-based emotion cues^21,22^ and are at risk for severe conduct problems across childhood and adolescence^23^. Thus, charting the intersection between emotion and language may improve our understanding of the development of childhood conduct problems and callous-unemotional traits, including whether, when, and how children use language to communicate about their emotions and social intentions.

Parents play a major role in their children’s socioemotional development^24^. While overly harsh or punitive parenting behaviors have been linked to childhood conduct problems^25,26^, parents also have a positive influence on children by scaffolding, shaping, and reinforcing socioemotional language understanding and expression^27,28^. For example, children whose parents use more emotional labeling during storytelling have higher scores on tests of emotion comprehension^29–31^. Likewise, children whose parents engage in more extensive and elaborative emotion language show greater empathy^32^, conscience development^33^, and social competence^34^. Taken together, these studies point to parent–child socioemotional language as a potentially fruitful avenue for intervention when children have conduct problems^35^. Parenting has also been linked to the development of callous-unemotional traits. For example, studies of early childhood have specifically linked callous-unemotional traits to low levels of parental warmth and affection^36,37^ and disrupted patterns of emotion socialization^38^, even after accounting for the severity of children’s conduct problems. Thus, an examination of parent–child language during emotion-evoking interactions could provide more granular insight into risk factors for both conduct problems and callous-unemotional traits.

Parental language is known to vary based on context (e.g., toy play vs. storytelling)^39^. Moreover, the time that parents and children spend together simply interacting may be just as important to socioemotional development as the linguistic content of those interactions^39,40^. For example, in addition to language, parent–child interactions typically encompass a multitude of nonverbal social bonding behaviors that have been linked to the development of social and behavioral difficulties in children^41^. Prior studies of parent–child language and conduct problems, however, are limited by a reliance on language data collected using observational designs and within laboratory-based or artificial contexts, which reduces ecological validity^42–44^. Few prior *experimental* studies of parent–child language interactions have been conducted in naturalistic contexts (e.g., the home environment) to control for non-linguistic (i.e., social or affiliative) features of parent–child interactions.

The current experiment addresses these knowledge gaps by measuring language use in parent–child dyads in one of two home-based play contexts, and testing links between language and child conduct problems and callous-unemotional traits. Parent–child dyads were randomized to receive either a socioemotional skills game (“social context”) or a mathematics game (“nonsocial context”) by mail. They were asked to play the game four times and parents uploaded an audio recording each time they played the game. Our linguistic analysis focused on words with socioemotional (e.g., love, happy, nervous, and mad), cognitive (e.g., think, know, and can), and mathematical (e.g., one, two, and three; see Supplemental Materials for more information) content. We included socioemotional and cognitive content because emotion recognition, social bonding, and perspective-taking are core difficulties among children with conduct problems and/or callous-unemotional traits^16,20^. We included mathematical content to serve as a comparison condition that we did not expect to be relevant to conduct problems or callous-unemotional traits. In our first aim, we assessed the proportion of each word type used by children and parents in each context. We hypothesized that dyads would produce more socioemotional and cognitive words in the social context and more mathematical words in the nonsocial context. In our second aim, we examined word use alignment (i.e., correlation between use of word types) between parents and children, hypothesizing greater alignment for socioemotional and cognitive words in the social context and greater alignment for mathematical words in the nonsocial context. Finally, under our third aim, we examined links between word production and parent–child word alignment across contexts and child conduct problems and callous-unemotional traits, hypothesizing that conduct problems and callous-unemotional traits would be related to lower socioemotional and cognitive word production and alignment, specifically in the social context.

## Results

Descriptive statistics and bivariate correlations for study variables are presented in Table S1. Our randomization was successful with no significant differences between experimental groups (i.e., social vs. nonsocial) on the basis of demographic characteristics (Table S2). Overall, the total number of words produced by parent–child dyads (*N* = 79; 75 mothers, 4 fathers; child age, *M* = 5.4 years, *SD* = 0.50, *range* = 5–6 years; 47 Female, 32 Male; 7 Asian, 31 Black, 38 White, and 3 “Other”) did not differ between the social versus nonsocial context (total words social, *M* = 433.65, *SD* = 199.58, *range* = 133–1097; total words nonsocial, *M* = 446.09, *SD* = 270.01, *range* = 119–1717; comparison, *t*(71.81) = .23, *p* = .82). Individually, parents did not differ in the number of words produced based on context (parents’ total words social, *M* = 632.96 vs. parents’ total words nonsocial, *M* = 572.96; *t*(75.11) =  − .84, *p* = .40). However, children produced more words in the nonsocial context than in the social context (children total words social, *M* = 234.35 vs. children total words nonsocial, *M* = 319.22; *t*(64.72) =  − 2.001, *p* = .048). Generally, parents spoke more words than children in both contexts (parents social, *M* = 632.96 vs. children social, *M* = 234.35, *t*(54.94) =  − 7.83, *p* < .001; parents nonsocial, *M* = 572.96 vs. children nonsocial, *M* = 319.23, *t*(67.62) =  − 3.89, *p* < .001).

Across both contexts, more than 70% of all words spoken by parents and children were content or function words (i.e., pronouns, determiners, prepositions, and adjectives). In terms of our substantive focus, socioemotional words comprised 2.7% of the total words produced by children and 3.1% of total words produced by parents. Cognitive words comprised 8.03% of words spoken by children and 9.1% of words spoken by parents, and mathematical words comprised 20.8% of words spoken by children and 10.2% of words spoken by parents.

### Aim 1: context modulates language content produced during parent–child interactions

**Figure 1:**
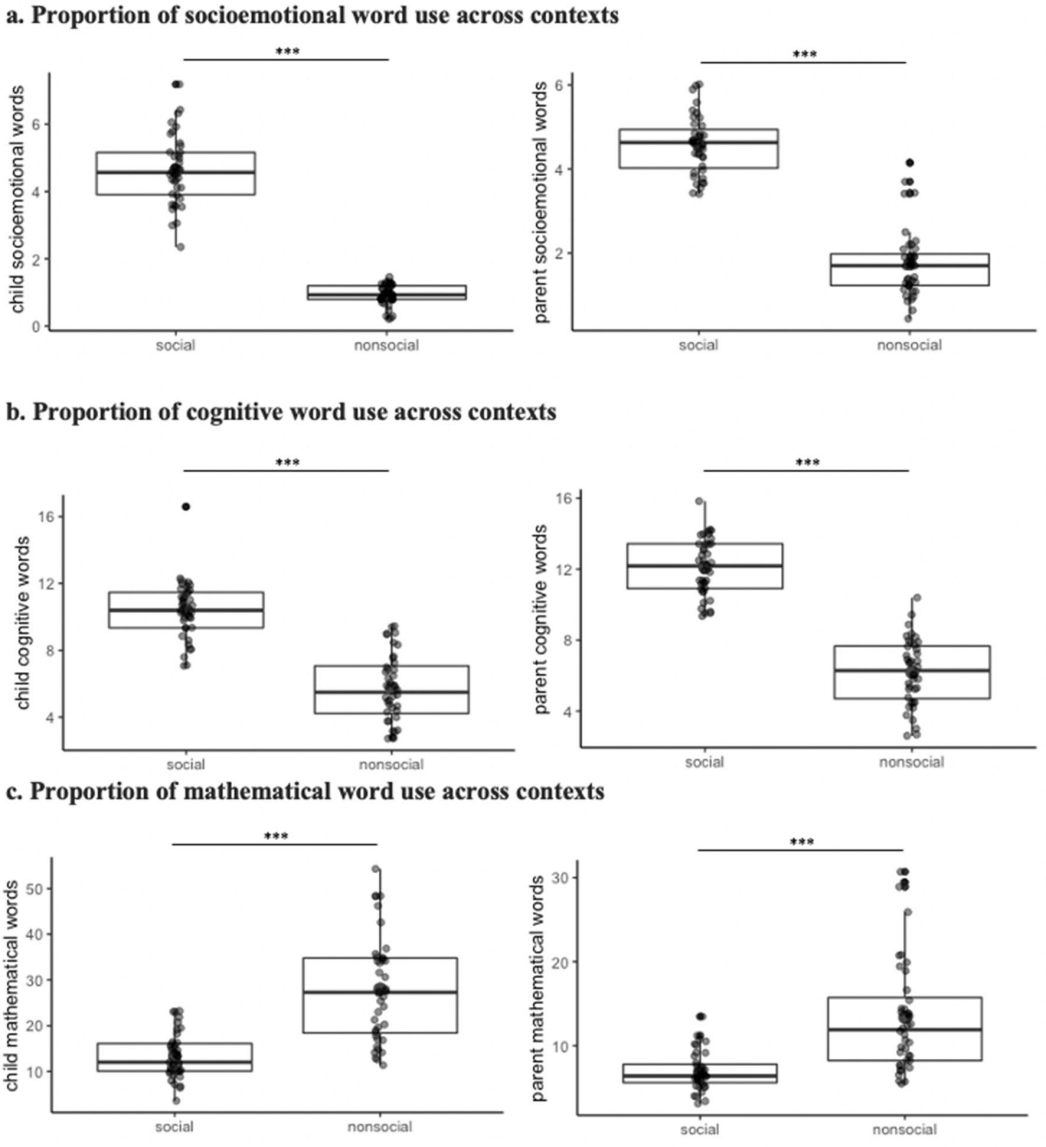
Proportion of speech produced by parents and child varies based on context. (**a**) Children, t(44.95) = 21.613, *p* < .001, d = 4.89, 95% CI = [3.358–4.048]; parents: t(75.88) = 16.62, *p* < .001, d = 1.93, 95% CI = 2.49–3.16. (**b**) Children, t(75.84) = 10.87, *p* < .001, d = 2.44, 95% CI = [3.81–5.52]; parents, t(75.97) = 14.92, *p* < .001, d = 3.35, 95% CI = [5.12–6.70]. (**c**) Children, t(53.43) = 7.36, *p* < .001, d =  − 1.65, 95% CI = [10.60–18.54]; parents, t(48.02) = 5.74, *p* < .001, d = 1.28, 95% CI = [4.19–8.70]. The red dot indicates the group mean.

As hypothesized, children and parents both produced more socioemotional words in the social context compared to the nonsocial context (children, *t*(44.95) = 21.613, *p* < .001, *d* = 4.89, 95% CI = [3.358–4.048]; parents: *t*(75.88) = 16.62, *p* < .001, *d* = 1.93, 95% *CI* = 2.49–3.16]; Fig. 1A). Both children and parents also produced more cognitive words in the social context than the nonsocial context (children, *t*(75.84) = 10.87, *p* < .001, *d* = 2.44, 95% CI = [3.81–5.52]; parents, *t*(75.97) = 14.92, *p* < .001, *d* = 3.35, 95% CI = [5.12–6.70]; Fig. 1B). Children and parents also produced more mathematical words in the nonsocial context (children, *t*(53.43) = 7.36, *p* < .001, *d* =  − 1.65, 95% CI = [10.60–18.54]; parents, *t*(48.02) = 5.74, *p* < .001, *d* = 1.28, 95% CI = [4.19–8.70]; Fig. 1C). Thus, our experimental manipulation of a naturalistic context (i.e., social vs. nonsocial games) produced distinct patterns of linguistic content among parent–child dyads.

As a post hoc test of convergent validity, we examined whether children’s emotional verbal fluency (i.e., number of emotion words freely produced by the child, a score generated by a separate task as described under Emotional Verbal Fluency), which had been collected during a separate assessment, was related to the proportion of different word categories produced by children in each context. We found that emotional verbal fluency was more strongly associated with the proportion of socioemotional words that children produced in the social context (interaction, *b* = .20, *t* = 2.33, *p* = .02; social context, *b* = .21, *t* = 3.34, *p* = .001; nonsocial context, *b* = .11, *t* = 2.61, *p* = .01). However, context did not moderate the relationship between emotional verbal fluency and cognitive or mathematical word production (see Table S3).

### Aim 2: context modulates language alignment during parent–child interactions

**Figure 2:**
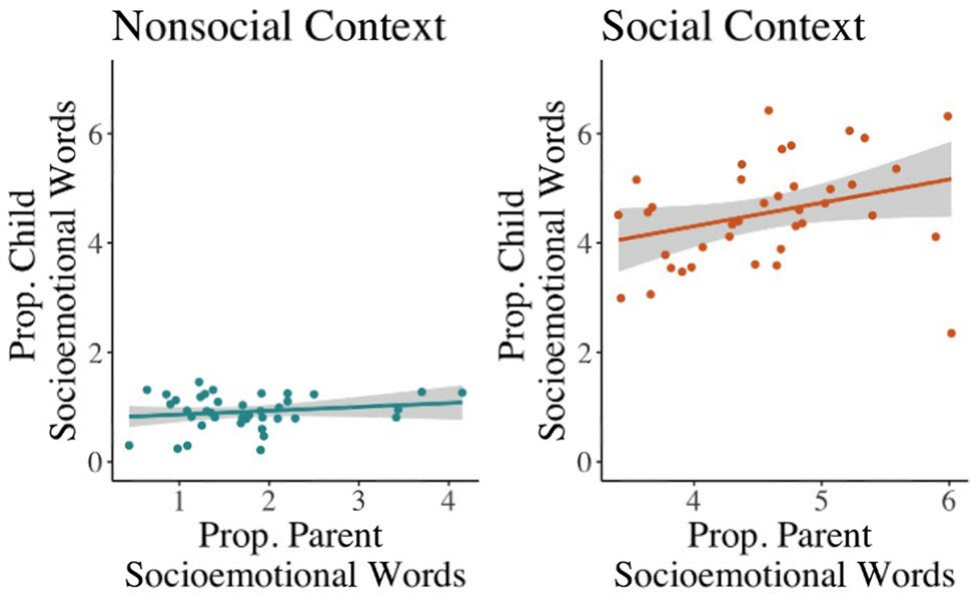
Parent–child socioemotional word use is aligned in the social but not the non-social context. The proportion of parent socioemotional words was related to the proportion of child socioemotional words only in the social context (interaction: b = .43, t = 1.98, *p* = .05; simple slope in social context, b = .44, t = 2.66, *p* = .01 vs. nonsocial context, b = .01, t = .09, *p* = .93).

Consistent with hypotheses, the proportion of socioemotional words produced by parents was aligned (i.e., positively correlated) with the proportion of socioemotional words produced by their children, but only in the social context (interaction, *b* = .43, *t* = 1.98, *p* = .05; social context, *b* = .44, *t* = 2.66, *p* = .01; nonsocial context, *b* = .01, *t* = .09, *p* = .93) (Fig. 2). In contrast to hypotheses, context did not moderate alignment in parent–child cognitive or mathematical word production (*ps* > .5; Table S4).

### Aim 3: Associations between context, parent–child language, and child conduct problems and callous-unemotional traits

**Figure 3:**
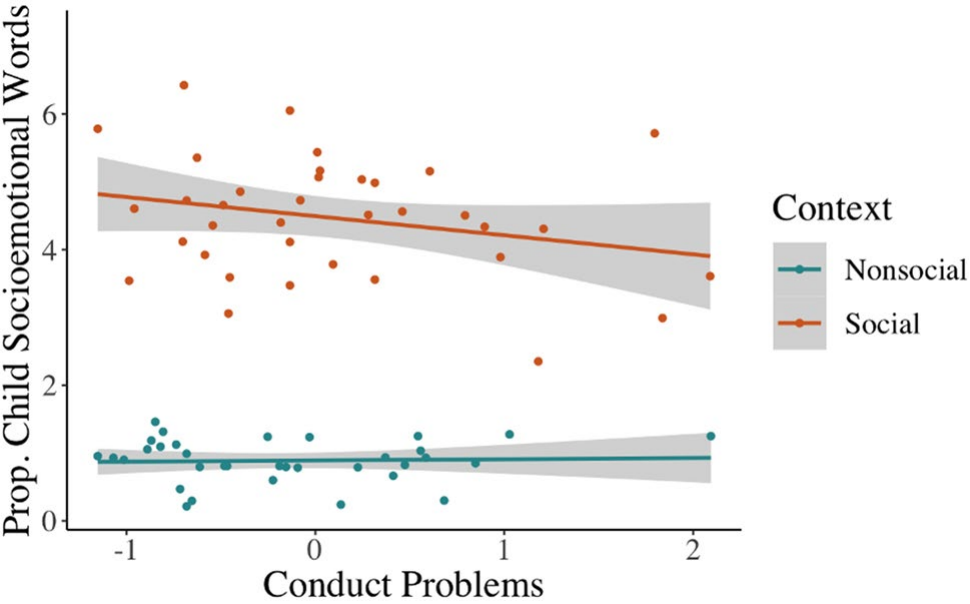
Child conduct problems were related to lower socioemotional word production in the social but not the nonsocial context. Children with higher conduct problems expressed fewer socioemotional words only in the social context (b =  − .30, t =  − 2.72, *p* = .009) but not the nonsocial context (b = .09, t = 0.51, *p* = .61).

Results revealed an interaction between conduct problems and context (*b* =  − .55, *t* =  − 2.51, *p* = .02), such that higher levels of child conduct problems related to children using fewer socioemotional words specifically in the social context (*b* =  − .40, *t* =  − 2.79, *p* = .007; Fig. 3), but not in the nonsocial context (*b* = .15, *t* = 0.76, *p* = .45). In contrast to hypotheses, there was no relationship between the proportion of socioemotional words produced and callous-unemotional traits in either context. However, there was a main effect of callous-unemotional traits on cognitive words, such that children with higher callous-unemotional traits produced fewer cognitive words during both the social and nonsocial contexts (*b* =  − .07, *t* =  − 2.09, *p* = .04; Table S5). There were no main effects or interactions with context for callous-unemotional traits or conduct problems in relation to the proportion of mathematical words produced by children. There were also no effects or interactions with child callous-unemotional traits or conduct problems for the proportion of socioemotional, cognitive, or mathematical words expressed by the parent (Table S6). Finally, while a significant three-way interaction suggested that parent–child language alignment may have been moderated by conduct problems (*b* =  − .67, *t* =  − 2.16, *p* = .04), neither of the follow-up two-way interactions reached statistical significance (alignment-by-conduct problems in the social context, *b* =  − .40, *t* =  − 1.95, *p* = .056; alignment-by-conduct problems in the nonsocial context, *b* = .27, *t* = 1.18, *p* = .24; Table S7). Callous-unemotional traits did not significantly moderate the degree of alignment of parent–child language for the proportion of socioemotional, cognitive, or mathematical words used across contexts (Table S8).

## Discussion

We measured parent–child language produced during social and nonsocial gameplay, and documented connections between language content, context, and child conduct problems and callous-unemotional traits. First, we showed that both children and parents used different language according to the context^45,46^, producing more socioemotional and cognitive words when playing a socioemotional skills game and producing more mathematical language when playing a mathematics-focused game. Additionally, children’s general emotional verbal fluency (measured independently from game play) was associated with children using more socioemotional words during parent–child interactions, indicating that the speech produced during game play reliably indexed children’s broader socioemotional expressive ability. Together, results illustrate the feasibility of manipulating a play context to provoke different types of word production in parents and children^47^. Importantly, we also demonstrated the feasibility of assessing language naturally in participants’ homes with very minimal prompting (i.e., written instructions that were mailed home) and without the actual physical or virtual presence of a research assistant^48–50^.

Second, we showed that context specifically modulated the alignment of parent and child word production, though only for socioemotional words and not for cognitive and mathematical word production. The presence of alignment in socioemotional word expression suggests that teaching parents to use socioemotional language could potentially have downstream effects on children’s use of socioemotional language (note, however, that we cannot assume directionality based on the current study)^51^. Most importantly, our findings suggest that context plays a causal role on the prevalence of socioemotional language produced by parents and children, and that social contexts explicitly facilitate more socioemotional language and greater alignment than nonsocial contexts. This pattern of findings provides supportive evidence for using targeted settings or paradigms to develop more effective family- or parent-based interventions to promote child social and emotional understanding and enhance socioemotional development and regulation.

Third, we found that children with more conduct problems produced fewer socioemotional words, specifically within the social context. This finding adds to research showing links between conduct problems and language difficulties more broadly^13,52^ and provides evidence for a specific connection between child conduct problems and socioemotional language, but not between conduct problems and child cognitive or mathematical word production. The ability to convey thoughts, feelings, and social intentions via language is vital to effective interpersonal connections, social bonding, and emotional resonance^53,54^, which are processes that are disrupted in children with conduct problems^11,13^. In contrast to our original hypotheses, context did not moderate the relationship between callous-unemotional traits and socioemotional word production. However, children higher in callous-unemotional traits produced a smaller proportion of cognitive words across both contexts. On one hand, this relationship contradicts some evidence that callous-unemotional traits are associated with intact perspective-taking and cognitive understanding of the world^55,56^. On the other hand, the cognitive word category in LIWC (our language analysis software) included words, such as *think* and *feel*, which are meaningful in the context of the difficulties experienced among children with high callous-unemotional traits, including lack of sensitivity towards or responding to the needs of others^57,58^, and our findings are consistent with meta-analytic work that directly linked callous-unemotional traits to lower cognitive empathy^20^.

Taken together, our findings have implications for etiological models and intervention targets for conduct problems and callous-unemotional traits with a particular focus on understanding and expressing socioemotional language, beginning much earlier in childhood^59–61^. Examples of this type of approach include interventions that strengthen positive parent–child interactions, promote communication skills in parents, and directly teach children better social and emotional communication skills, all of which have been associated with reductions in child conduct problems and increases in more constructive parent and child relationships in prior literature^30,48,49,62,63^. The association between callous-unemotional traits and lower cognitive word production highlights an important target for intervention through a focus on language during parent–child interactions, with preliminary evidence that adjunctive treatment modules tailored to the specific cognitive language features of children with high callous-unemotional traits can be effective in directly reducing their callous-unemotional traits, while increasing empathy^64,65^.

Our results should be interpreted in light of several important limitations. First, the current analysis cannot speak to directionality in the relationship between parent and child language production, as language was analyzed at the conversation level rather than at the turn level, across all four recordings. Future research is necessary to chart, with greater granularity, the dynamics of parent–child conversations, including quantifying who initiates the use of socioemotional language, the latency of parents or children to respond to socioemotional bids, and the possibility that children use fewer socioemotional words because parents initiate them less to begin with (or vice versa)^66,67^. Second, we analyzed proportions of socioemotional words produced relative to total words, which was derived by computing an average of the proportion of emotion and social behavior words. Within the LIWC dictionary, there was some overlap of these categories (e.g., love and good appear under both categories), which meant there may have been overrepresentation of the overlapping words within our socioemotional word production variable. Nevertheless, results from examining emotion and social word categories separately were similar, as presented in Supplemental Materials. Notably, the LIWC is just one available method to categorize words, and future studies of parent–child interactions are needed to leverage the recent and dramatic changes in natural language processing algorithms within artificial intelligence^68^. Third, we recruited a small community sample with relatively low levels of conduct problems and callous-unemotional traits (i.e., *n* = 10 had scores ≥ 4, established as being within the clinical range for the SDQ conduct problems scale)^69,70^. Thus, while similar associations might be hypothesized in samples exhibiting greater conduct problem severity, future research is warranted to replicate our findings in larger samples that include more children with clinically significant conduct problems. Moreover, subsequent studies, properly powered, have the potential to reveal novel linguistic features that could serve both as risk markers and treatment targets for parent–child interventions aimed at fostering socioemotional development in children with conduct problems and/or high callous-unemotional traits. Fourth, the contexts were not equivalent, with children in the social condition playing four different socioemotional games and those in the nonsocial condition playing the *same* math game four times. At the same time, socioemotional functioning represents a complex constellation of skills, as captured within the four social games (i.e., empathy, emotions, manners, and friendship), which may have enhanced the validity of our approach. Moreover, there was no effect of the order in which the social games were played on word production. Finally, although the racial diversity within our sample was representative of the city in the northeastern United States from which we recruited, parents were otherwise highly educated and our sample, more broadly, could be categorized as WEIRD (i.e., Western, educated, industrialized, rich and democratic) and not generalizable to all populations^71,72^. Future studies are needed that are powered and designed to evaluate parental scaffolding and/or use of socioemotional language across different cultures.

In sum, our findings contribute to a growing literature focused on socioemotional language interactions between parents and children during early childhood, provide additional evidence for the critical role of social context in promoting certain types of language expression, and give preliminary evidence for links between parent–child socioemotional language and conduct problems in children. Through an experimental design that randomized parent–child dyads into one of two conditions, we showed that language differed significantly between contexts, while keeping constant the semi-structured nature of the parent–child interaction (i.e., the two “game-playing” situations). Our approach highlights the utility of leveraging brief, naturalistic home-based approaches, which minimize demands on families. This point is salient in the context of the COVID-19 pandemic, which precipitated a new “normal” centered on virtual encounters, with some parents preferring online assessments or services for pediatrician visits^73^ or parenting support^74,75^. Indeed, an important takeaway from this study is the high rate of acceptability from parents for uploading recorded samples of interactions between themselves and their children. This willingness to share parent–child recordings on a secure platform opens the door for future distanced, virtual, or asynchronous assessments and interventions. Such options may be more accessible for families, particularly those living in more rural/remote areas or unable to attend in-person sessions due to socioeconomic, family, or employment constraints, while potentially generating greater insights into parent–child interactions that are more naturalistic than might otherwise be obtained through lab-based assessments or interventions^76–78^.

## Methods

### Participants

Participants were 79 children aged 5–6 years old (*M*_age_ = 5.4 years, *SD*_age_ = .50; 47 Female, 32 Male; 7 Asian, 31 Black, 38 White, and 3 “Other”) and parents (75 mothers, 4 fathers) recruited from a city in the northeastern United States. 54.4% of parents reported having a graduate-level degree, 30.4% had a bachelor-level degree, 7.6% had some college but no degree, 2.5% had an associates-level qualification, 3.8% had a high school qualification or less, and 1.3% declined to answer. The average monthly household income was $10,026 (*SD* = 9.236), although 21.5% of the sample reported an annual income that fell below the median for [masked for review] households based on the 2021 census (U.S. Census Bureau, 2021). The sample was recruited for the [name masked for review] study that involved multiple measures of emotional development, a subset of which are presented here. 12 additional participants were recruited into the study but were excluded prior to analysis because they did not upload any recordings (*n* = 9), the recordings were not clear enough to transcribe (*n* = 1), or participants did not follow the task instructions (e.g., played the game with multiple children, *n* = 2). We included the number of games completed as a covariate in analyses (*M* = 3.59, SD = .809, range = 1–4).

### Design and procedure

As a part of the [name masked for review] study, participants were randomly assigned to one of three experimental contexts: (1) Mailed a socioemotional game to play at home (“social context”), (2) Mailed a mathematical game to play at home (“non-social context”), or (3) Received no game to play at home (“control”). The current study focuses on the words that parents and children produced while playing the games, thus the control condition was not included in our data analysis. For the two experimental contexts, parent–child dyads were asked to play the game four times during a 6–8-week period at a time of the day when there would be minimal external distractions. They were asked to audio record each time they played the game using a voice recording application (e.g., “voicenote”), which parents then uploaded to a secure website (www.SendSafely.com).

Participants played the games at their own pace in any order and were instructed that there was no time limit. Accordingly, there was significant variation in the length of the uploaded recordings and the numbers of words produced by children and parents (see “Results” section). Audio recordings were transcribed by the first, fifth, and sixth authors. We double transcribed a randomly selected 25% of recordings to monitor reliability and accuracy (99.96% for social context; 98.70% for nonsocial context). Transcriptions were analyzed using the Linguistic Inquiry and Word Count (LIWC) software, which automatically produced an output of the proportion of words used in different categories (see “Language data” section for more information). Prior to randomization (baseline) and after the 6–8-week study period (follow-up), parents completed a series of surveys using Qualtrics, including completing the questionnaires used in the current study. In addition, at baseline and follow-up, parents and children completed an online “virtual visit” (over Zoom) with a trained research assistant. During this visit, children completed an emotional verbal fluency test. Informed consent for themselves and their child was obtained at the beginning of each Zoom visit from parents who provided an electronic signature. Informed verbal assent was obtained from children. All procedures were approved by the University of Pennsylvania Institutional Review Board and all methods were performed in accordance with the relevant guidelines and regulations.

#### Social context

Participants randomized to the social context (*n* = 39) were mailed the game, *Social Skills Board Games* (*JRL426 by Junior Learning*), which included four separate board games relating to socioemotional skills: empathy, friendship, manners, and emotions. Having four games allowed us to capture the complexity of socioemotional skills, and the games were designed to promote the use of each specific skill, which are core to socioemotional abilities that are disrupted among children with conduct problems^16,20,25^. For each game, participants took turns moving their piece forward on a board following a die roll. Players read the scenario on which their piece landed and then spun a spinner, which had a question unique to each of the games. The player responded with a statement relating the scenario and the spinner, and if both players agreed to the statement, the player stayed on the landing spot until their next turn. Players continued to take turns until someone reached the end of the board and won (see Supplemental Materials for game instructions). Parents were instructed to take the time to read the instructions and explain how the task worked to the child before starting the game. The parent and child were asked to play each one of the four games once, in any order, over a 6–8-week period, which would ensure the right balance of novelty and having enough time to capture expressed language without being overly burdensome to participants. Participants audio recorded the game and uploaded recordings to a secure website. There was variability in the order of games (Table S9) and game-related differences for all word types (Table S10; though there was no effect of the order in which the social games were played on word production). In general, the richest dialogue was elicited in the Emotions game and the least rich dialogue was elicited in the Manners game (see Supplemental Materials for more descriptive data across socioemotional board games and Table S11 for pairwise comparisons).

#### Non-social context

Participants randomized to the non-social condition (*n* = 40) were mailed the game, *Cat Owl* (*Terribly Interesting Game! Mental Calculation by The Brainy Band*), which was a mental arithmetic card game. The parent and child were given a deck of cards, which had pictures of animals with different attributes. One player picked a card and rolled a die, which had names of each attribute. Based on the attribute the player rolled, they stated the number of animals on the card that had the attribute. If the player counted the correct number, they kept the card, and if not, the card was placed back in the stack. The players took turns until all the cards were gone, and the person with the greatest number of cards won the game (see Supplemental Materials for game instructions). The parent and child were asked to play the game four times over 6–8 weeks, audio record the game, and upload recordings to a secure website.

### Measures

#### Conduct problems

We assessed conduct problems using two different measures, which were combined across the baseline and follow-up visits to provide a full index of childhood conduct problems encompassing aggression, rule violations, and disruptive behavior. First, parents completed the 32-item Subtypes of Antisocial Behavior (STAB) Questionnaire^79^, which assessed physically aggressive (e.g., assaulting and bullying others) and socially aggressive (e.g., excluding others and gossiping) behavior in children^80^. Items were rated on a 5-point scale (1 = never to 5 = nearly all the time). Second, parents completed the 5-item conduct problems scale from the Strengths and Difficulties Questionnaire (e.g., “fights with other children or bullies them,” “often lies or cheats,” and “steals from home, school or elsewhere”)^81,82^. Items were rated on a 3-point scale (0 = not true, 2 = certainly true). Since items were available from both baseline and follow-up, we computed a sum of the mean of items from each time point for both the STAB questionnaire and the conduct problems subscale of the SDQ^83,84^. Scores were highly correlated (*r* = .73, *p* < .001) and we combined them into a single conduct problems measure by computing a mean of the z-scores (the results for STAB and SDQ when analyzed separately are in the Supplemental Materials).

#### Callous-unemotional traits

We assessed callous-unemotional traits using parent reports on the Inventory for Callous-Unemotional Traits (ICU ^85^;), a 24-item measure assessing callousness (e.g., “is concerned about the feelings of others”), uncaring (e.g., “feels bad or guilting when he/she has done something wrong”), and unemotionality (e.g., “expresses his/her feelings openly”)^86,87^. Consistent with prior studies^88^, we excluded items 3 and 10 from the total callous-unemotional traits score. We computed total scores by summing the mean item ratings from data collected at baseline and follow-up^83,84^.

#### Emotional verbal fluency

We assessed emotional verbal fluency using a previously validated task^89^ where children were asked to list all the emotions/feelings words they knew in a 30 s period during online Zoom visits *(“Can you tell me all the emotion or feeling words you know? So different words for how you are feeling in your heart? Ready? Go”*). Responses were video recorded and then each unique emotion or feeling word produced by the child was coded by trained research assistants and summed to generate a total emotional verbal fluency score. Scores from baseline and follow-up visits did not differ (*t*(145.98) =  − 1.34, *p* = .18), and we used children’s higher score from either the baseline or follow-up visits in analyses.

#### Language data

To avoid inconsistency in linguistic coding generated by human coders, we processed the transcribed language data using the 2022 version of Linguistic Inquiry and Word Count (LIWC)^67,90^. LIWC is a text analysis system that classifies words according to their definitions in the LIWC dictionary and was specially designed for language researchers to associate significant psychosocial concepts and theories with language elements, such as words, phrases, and linguistic structures^91^ (see Supplemental Materials for more detailed description). For analyses, we used the total word count and four linguistic categories: emotion words (e.g., happy, hope, good) were from the broader LIWC category of affect and included both positive and negative emotion words; social behavior words (e.g., said, care, love) included words centered on politeness, prosocial behavior, conflict, and communication, forming a subcategory of the broader social processes LIWC category; cognitive processes words (e.g., believe, think, know) came from the broader LIWC category of cognition; and mathematical words were those that encapsulated any number-related words spoken. To create a composite of socioemotional speech, we used an average of the emotion and social behavior categories (the results showed very similar patterns for the categories separately, see Supplemental Materials). LIWC calculated the proportion of words in the text that fell into a predetermined category by dividing the number of words in each category by the total number of words produced. Thus, higher scores in any category indicated that the speaker produced a greater proportion of those words during the game play.

### Analysis plan

To address our first aim, we compared the proportion of socioemotional, mathematical, and cognitive word produced in the social and non-social contexts for children and parents (two-tailed t-tests, Bonferroni corrected for multiple comparisons). A test of proportions suggested that there was an equivalent number of dyads who completed all 4 games in the social and nonsocial conditions (68% in the nonsocial condition vs. 87% in the social condition; X2(1) = 3.3, *p* = .07). After averaging the proportion of (socioemotional/cognitive/mathematical) words used during each game, we divided the number of words produced by the number of games played to create the final proportion per context and minimize data loss for those who did not play the game all four times. As a post hoc test of convergent validity, we also tested whether context moderated the relationship between child emotional verbal fluency and words used, with separate models for socioemotional, cognitive, or mathematical words. Specifically, we conducted a linear regression, regressing the proportion of words produced by the child on context (non-social =  − .5, social = .5), emotional verbal fluency score (mean-centered), and their interaction. We included the proportion of emotion words produced by the parent, child age, child gender, child race, and number of games completed as covariates. To address our second aim focused on alignment of parent and child word production, we regressed the proportion of words produced by the child, the proportion of different words produced by the parent (mean-centered), and their interaction on context (social vs. non-social). We ran separate models for socioemotional, cognitive, and mathematical words. As before, we controlled for parent, child age, child gender, child race, and number of games completed, as well as emotional verbal fluency. Finally, to test our third aim, we examined whether context moderated the relationship between word production and both child conduct problems and callous-unemotional traits. We regressed the proportion of words produced by the child, conduct problems, callous-unemotional traits, the interactions of context with conduct problems and callous-unemotional traits, and the interaction between conduct problems and callous-unemotional traits, with separate models for parent and child socioemotional, cognitive, and mathematical words, as well as their alignment. Interaction terms were mean-centered, and we included the covariates listed above.

### Transparency and openness

De-identified datasets and analysis scripts are on Open Science Framework (https://osf.io/ytzmn/?view_only=a0d03ffcbfc8493f98b8246a3f9135ed). We used R version 2022.02.1 for analyses (R Core Team, 2019), including the tidyverse package^92^ for data organization, lme4 package^93^ for linear models, and ggplot2^94^ for visualization. We reported how we determined our sample size, all data exclusions, and all manipulations and measures in the study. The study design and analyses were not pre-registered.

### Supplementary Information

Supplementary Information.

## Data Availability

De-identified datasets and analysis scripts are on Open Science Framework (https://osf.io/ytzmn/?view_only=a0d03ffcbfc8493f98b8246a3f9135ed).
