# Supplementary material for: Childhood conduct problems and parent–child talk during social and nonsocial play contexts: a naturalistic home-based experiment
Source: Sci Rep. 2024 Jan 10;14:1018. doi: 10.1038/s41598-024-51656-w (PMC10781972; doi:10.1038/s41598-024-51656-w)
Supplement: Supplementary file 1 — Supplementary Information. [file 41598_2024_51656_MOESM1_ESM.docx]

**Supplemental Materials**

**Childhood Conduct Problems and Parent-Child Talk During Social and Nonsocial Play Contexts: A Naturalistic Home-Based Experiment.**

Sydney Sun^1^, Rista C. Plate^1^, Callie Jones^1^, Yuheiry Rodriguez^1^, Chloe Katz^1^, Melissa Murin^1^, Jules Pearson^1^, Julia Parish-Morris^2,3,4^ & Rebecca Waller^1^*

^1^Department of Psychology, University of Pennsylvania, Levin Building, 425 S. University Ave., Philadelphia, PA 19104

^2^Center for Autism Research, Children's Hospital of Philadelphia, Philadelphia, PA, USA.

^3^Department of Child and Adolescent Psychiatry and Behavioral Sciences, Children's Hospital of Philadelphia, Philadelphia, PA, USA

^4^Department of Psychiatry, Perelman School of Medicine, University of Pennsylvania, Philadelphia, PA, USA

*rwaller@sas.upenn.edu

Contents

[Board Game Instructions by Context 2](#_Toc149070366)

[Social Context 2](#_Toc149070367)

[Manners Game 2](#_Toc149070368)

[Friendship Game 2](#_Toc149070369)

[Empathy Game 3](#_Toc149070370)

[Emotions Game 3](#_Toc149070371)

[Nonsocial Context 3](#_Toc149070372)

[Descriptive Data Comparing Time Spent Playing Social versus Nonsocial Games 4](#_Toc149070373)

[Linguistic Inquiry and Word Count (LIWC) 5](#_Toc149070374)

[Results for Emotion and Social Behavior Word Categories Examined Separately 6](#_Toc149070375)

[Results for Two Conduct Problem Measures When Examined Separately 7](#_Toc149070376)

[Table S1. Descriptive Statistics and Bivariate Correlations 8](#_Toc149070377)

[Table S2. Comparison of Demographic Characteristics between Participants in Randomly-Assigned Contexts 9](#_Toc149070378)

[Table S3. Full Model Output for Relationships Between Emotional Verbal Fluency and Proportion of Child Word Production 10](#_Toc149070379)

[Table S4. Full Model Output for Relationships Between Child and Parent Word Production (“Alignment”) Across Contexts 11](#_Toc149070380)

[Table S5. Full Model Output for Relationships between Conduct Problems, Callous-Unemotional Traits, and Child Word Production 12](#_Toc149070381)

[Table S6. Full Model Output for Relationships between Child Conduct Problems, Callous-Unemotional Traits, and Parent Word Production 13](#_Toc149070382)

[Table S7. Full Model Output for Relationships between Conduct Problems and Child and Parent Word Production (“Alignment”) Across Contexts 14](#_Toc149070383)

[Table S8. Full Model Output for Relationships between Callous-Unemotional Traits and Child and Parent Word Production (“Alignment”) Across Contexts 15](#_Toc149070384)

[Table S9. Frequencies of the 14 Orders in which the Social Games were Played 16](#_Toc149070385)

[Table S10. Mean Proportion of Number, Cognitive, and Socioemotional Words Produced in Individual Social Games 17](#_Toc149070386)

[Table S11. Social Board Games Pairwise Comparisons 18](#_Toc149070387)

# Board Game Instructions by Context

Participants were randomly assigned to one of three experimental contexts: (1) Mailed a socioemotional game to play at home (“social context”), (2) Mailed a mathematical game to play at home (“non-social context”), or (3) Received no game to play at home (“control”). The current study focuses on the words that parents and children produced while playing the games, thus the control condition was not included in our data analysis. For the two experimental contexts, parent-child dyads were asked to play the game four times during a 6-8-week period at a time of the day when there would be minimal external distractions. They were asked to audio record each time they played the game using a voice recording application (e.g., “voicenote”), which parents then uploaded to a secure website (www.SendSafely.com). Participants played the games at their own pace and were instructed that there was no time limit. Accordingly, there was significant variation in the length of the uploaded recordings and the numbers of words produced by children and parents (see Results). Audio recordings were transcribed by the first, fifth, and sixth authors. We double transcribed a randomly selected 25% of recordings to monitor reliability and accuracy (99.96% for social context; 98.70% for nonsocial context).

## Social Context

### Manners Game

1. Have both players choose their markers and place them on the start.

2. Pick one player to go first. Have that player roll the die to move forward.

3. After the player moves their piece forward that amount, that player should spin the spinner.

4. If the spinner lands on a direction, the player should move their piece accordingly.

5. If the spinner lands on a word, use that word to make a polite statement or question about

the situation on the space the player’s marker is on.

6. If the player can make a question or statement, they get to keep their new space on the

board.

7. If the player cannot make a question or statement, they have to go back to where they were

before their roll.

8. Switch players and continue until someone reaches the finish line.

### Friendship Game

1. Have both players choose their markers and place them on the start.

2. Pick one player to go first. Have that player roll the die to move forward.

3. After the player moves their piece forward that amount, that player should read the scenario

they landed on.

4. That player may then spin the spinner.

5. If the spinner lands on a direction, the player should move their piece accordingly.

6. If the spinner lands on a sentence, use that sentence to make a statement about the scenario

the player landed on.

7. If the other players agree with the statement, the player may keep the spot they’ve landed

on.

8. If the other players do not agree with the statement, the player has to go back to where they

were before their roll.

9. Switch players and continue until someone reaches the finish line.

### Empathy Game

1. Have both players choose their markers and place them on the start.

2. Pick one player to go first. Have that player roll the die to move forward.

3. After the player moves their piece forward that amount, that player should read the scenario

they landed on.

4. That player may then spin the spinner.

5. If the spinner lands on a direction, the player should move their piece accordingly.

6. If the spinner lands on a question, answer that question about the scenario the player landed

on.

7. If the other players agree with the statement, the player may keep the spot they’ve landed

on.

8. If the other players do not agree with the statement, the player has to go back to where they

were before their roll.

9. Switch players and continue until someone reaches the finish line.

### Emotions Game

1. Have both players choose their markers and place them on the start.

2. Pick one player to go first. Have that player roll the die to move forward.

3. After the player moves their piece forward that amount, that player should read the scenario

they landed on.

4. That player may then spin the spinner.

5. If the spinner lands on a direction, the player should move their piece accordingly.

6. If the spinner lands on an emotion, the player should act out the emotion in the scenario

that they landed on.

7. If the emotion fits the scenario and the player is able to act it out, the player may keep their

new spot on the board.

8. If the emotion does not fit the scenario and the player cannot act it out, the player must go

back to their previous spot on the board.

9. Switch players and continue until someone reaches the finish line.

## Nonsocial Context

1. Place the cards on the table, back side up. Remove the magic cards that only have one animal on them from the stack.

2. The youngest player starts first.

3. The first player turns the top card over and puts it in front of them.

4. The first player can then roll the dice with attributes (not numbers).

5. The first player will then say the number of animals on the card that have that attribute.

6. If the player is correct, they keep that card. If they are wrong, they place that card at the

bottom of the stack and the next player takes their turn.

7. The game continues until all cards are taken. The player at the end of the game with the

most cards wins.

# Descriptive Data Comparing Time Spent Playing Social versus Nonsocial Games

We regressed amount of time playing (in seconds) on time (first, second, third, or fourth time playing the game), condition (nonsocial = -.5, social = .5) and their interaction. Participants spent less time playing the social game versus the nonsocial game (b = -231.71, SE = 67.67, t = -3.42), and spent less time playing the games over time (b = -30.08, SE = 8.08, t = -3.72). These main effects were qualified by an interaction such that participants spent less time playing the nonsocial game over time as compared to the social game, which stayed relatively stable in game time (b = 43.74, SE = 16.15, t = 2.71). These results underscore the importance of examining proportions of speech rather than raw word production to account for variability in game play time.

# Linguistic Inquiry and Word Count (LIWC)

All transcriptions were coded by the Linguistic Inquiry and Word Count (LIWC) text analysis software. LIWC aims to provide insights into the psychological, emotional, and cognitive aspects of a text by categorizing words into specific linguistic and psychological dimensions based on its own special dictionary [89]. The LIWC software uses this dictionary to analyze a given text by tokenizing the text into words and then classifies each word within its own dictionary. When a word is found in the dictionary, the software assigns it to one or more of the predefined categories or dimensions. The output of the analysis provides information on the presence and frequency of words within these categories in the text.

The development of LIWC’s dictionary involved a multi-step process [88]. It began with the conceptualization of linguistic and psychological dimensions (e.g., affective processes, social processes, etc.), and a wide array of words was selected to represent these dimensions, with input from psychologists to ensure accuracy [68]. Large datasets of text were analyzed, and words were manually categorized based on their relevance to predefined dimensions. Statistical validation tests were conducted to assess reliability, and once validated, the dictionary was implemented in LIWC software, facilitating automated text analysis. The dictionary continues to evolve with periodic updates to reflect changes in language use and improve categorization accuracy. We utilized LIWC’s most recent version (LIWC 2022) for our analyses.

Per LIWC’s dictionary definitions, the emotion category captures words related to emotional states and expressions (e.g., happy, sad, angry, and excited) and encompasses subcategories such as positive emotion, negative emotion, and anxiety. The social behavior category focuses on words associated with social interactions and relationships (e.g., friend, family, talk, and help) with subcategories like prosocial behavior, politeness, and communication. Cognitive processes category identifies words related to thinking and intellectual activities (e.g., think, consider, know, understand), incorporating subcategories like insight (e.g., know, how, think), causation (e.g., because, why), and differentiation (e.g., but, not, or). There was overlap as words may fit into multiple categories and was reflected as such in the LIWC outputs.

In our analyses, we found our reliability of transcripts to be 99.96% for social context and 98.70% for nonsocial context. These numbers were determined based on the LIWC output of double transcribing 25% of the recordings. We compared the differences in the proportions of social behavior, emotion, cognitive processes, and math words that LIWC provided for the double transcriptions in each context. We then averaged the proportion of similarity between the word categories within each context and achieved the output of 0.9996 and 0.9870 similarity in LIWC word output for the social and math contexts, respectively.

# Results for Emotion and Social Behavior Word Categories Examined Separately

Using the same analysis approach reported in the main manuscript, children and parents both produced a higher proportion of emotional and social behavior words in the social versus nonsocial context (children emotion: *t*(64.04)=19, p<.001, *d*=4.50, *95% CI*=[2.63–3.21]; parents emotion: *t*(62.02)=8.60, *p*<.001, *d*= 1.93, *95% CI*=[1.59 – 2.56]; children social behavior: *t*(44.53)=15.70, *p*<.001, d=3.55, *95% CI*=[3.91–5.06]; parents social behavior: *t*(73.64)=14.13, *p*<.001, *d*=3.18, *95% CI*=3.07 – 4.08]). Children’s emotional verbal fluency (i.e., the number of emotion words children could freely name in 30s) was related to children’s emotional word production (*b*=.14, *t*=4.04, *p*<.001), but not social behavior word production (*b*=.08, *t*=1.06, *p*=.29). Similarly, context moderated the relationship between children’s emotional verbal fluency and emotion word production (*b*=.25, *t*=3.56, *p*<.001), but not social behavior word production (*b*=.15, *t*=0.97, *p*=.34).

There was a stronger correlation between parent emotion word production and child emotion word production (*b*=.29, *t*=3.94, *p*<.001) as well as between parent social behavior word production and child social behavior word production (*b*=.38, *t*=3.14, *p*=.003). Context moderated the relationship for emotion words (*b*=.62, *t*=4.25, *p*<.001), but not social behavior words, though the pattern was in the same direction (*b*=.39, *t*=1.56, *p*=.12).

There was an interaction between conduct problems and context for emotion word production (*b*=-0.44, *t*=-3.19, *p*=.002), such that conduct problems were associated with lower emotion word production in the social, relative to the nonsocial, context. The interaction was not significant for social behavior words, but it followed the same pattern (*b*=-.41, *t*=-1.22, *p*=.23). There was no main effect of CP for emotion word production (*b*=.01, *t*=0.13, *p*=.89) or social behavior word production (*b*=-.14, *t*=-0.70, *p*=.49).

# Results for Two Conduct Problem Measures When Examined Separately

We assessed conduct problems using two different measures, which were combined across the baseline and follow-up visits to provide a full index of childhood conduct problems encompassing aggression, rule violations, and disruptive behavior. First, parents completed the 32-item Subtypes of Antisocial Behavior (STAB) Questionnaire, which assesses physically aggressive (e.g. assaulting and bullying others) and socially aggressive (e.g. excluding others and gossiping) behavior in children. Second, parents completed the 5-item conduct problems scale from the Strengths and Difficulties Questionnaire (e.g., “fights with other children or bullies them,” “often lies or cheats,” and “steals from home, school or elsewhere”). Since items were available from both baseline and follow-up, we took the higher-rated item from each time point for both the STAB and the conduct problems subscale of the SDQ. Scores were highly correlated (r = .73, p < .001) and we combined them into a single conduct problems measure by computing a mean of the z-scores (see **Results** in the main paper).

Results for STAB and SDQ were similar when analyzed separately with an interaction between STAB and context (*b*=-0.34, *t*=-2.12, *p*=.04) and SDQ and context (*b*=-0.35, *t*=-1.96, *p*=.05) for socioemotional word production, such that higher STAB and SDQ scores were associated with lower socioemotional word production in the social context (as compared with the nonsocial context). The relationships between STAB and word production and SDQ and word production were not significant for socioemotional, mathematical, or cognitive words (*ps* > .1) and context did not interact with STAB or SDQ for mathematical or cognitive words (*ps* > .2). The 3-way interaction between context, parent socioemotional word production and conduct problems showed a trend to significance for both the SDQ (*b*=-0.61, *t*=-2.01, *p*=.05) and STAB (*b*=-0.43, *t*=-1.75, *p*=.09). The 3-way interaction was not significant for mathematical or cognitive word production.

# Table S1. Descriptive Statistics and Bivariate Correlations

| Variable | *M* | *SD* | 1 | 2 | 3 | 4 | 5 | 6 | 7 | 8 | 9 | 10 |
| --- | --- | --- | --- | --- | --- | --- | --- | --- | --- | --- | --- | --- |
|  |  |  |  |  |  |  |  |  |  |  |  |  |
| 1. Child Socioemotional Words | 2.74 | 2.01 |  |  |  |  |  |  |  |  |  |  |
| 2. Child Mathematical Words | 20.78 | 11.47 | -.58** |  |  |  |  |  |  |  |  |  |
| 3. Child Cognitive Words | 8.03 | 3.02 | .76** | -.80** |  |  |  |  |  |  |  |  |
| 4. Conduct Problems | 0.01 | 0.93 | .10 | -.12 | .09 |  |  |  |  |  |  |  |
| 5. CU Traits | 15.36 | 6.95 | -.15 | .22 | -.25* | .46** |  |  |  |  |  |  |
| 6. Child Age | 5.93 | 0.52 | .21 | -.10 | .04 | .13 | -.12 |  |  |  |  |  |
| 7. Emotional Verbal Fluency | 4.72 | 1.93 | .18 | .03 | .03 | .06 | -.03 | .20 |  |  |  |  |
| 8. Parent Socioemotional Words | 3.14 | 1.61 | .86** | -.54** | .70** | .17 | -.11 | .11 | .13 |  |  |  |
| 9. Parent Mathematical Words | 10.20 | 5.97 | -.48** | .72** | -.63** | -.09 | .13 | .08 | .05 | -.49** |  |  |
| 10. Parent Cognitive Words | 9.10 | 3.45 | .78** | -.65** | .80** | .17 | -.12 | .06 | -.01 | .77** | -.69** |  |
| 11. Number of Recordings | 3.59 | 0.81 | .25* | -.08 | .08 | -.20 | -.02 | .08 | -.02 | .18 | -.12 | .20 |

*Note.* *indicates *p* < .05. ** indicates *p* < .01. Means/standard deviations for word categories represent proportion of words produced.

# Table S2. Comparison of Demographic Characteristics between Participants in Randomly-Assigned Contexts

|  | **Social Context**  (*n=*39)  *M (SD)* | **Nonsocial Context** (*n=*40*)*  *M (SD)* | Test of Comparison Between Groups |
| --- | --- | --- | --- |
| Child Age | 5.99 (.52) | 5.87 (.52) | *t* = 1.04, *p* = .30 |
| Annual Income | 123198.17 (81666.92) | 116730.26 (133234.30) | *t* = 0.28, *p* = .77 |
| Emotional Verbal Fluency | 4.87 (1.79) | 4.58 (2.06) | *t* = 0.68, *p* = .49 |
| Conduct Problems | .18 (1.07) | .16 (.73) | *t* = 1.53, *p* = .13 |
| CU Traits | 14.50 (6.51) | 16.23 (7.36) | *t* = -1.04, *p* = .30 |
| Child Race | 4 Asian, 13 Black, 21 White, 1 "Other" | 3 Asian, 18 Black, 17 White, 2 "Other" | *X^2^* = .61, *p* = .43 |
| Child Gender | 14 M, 25 F | 18 M, 22 F | *X^2^* = .35, *p* = .55 |

# Table S3. Full Model Output for Relationships Between Emotional Verbal Fluency and Proportion of Child Word Production


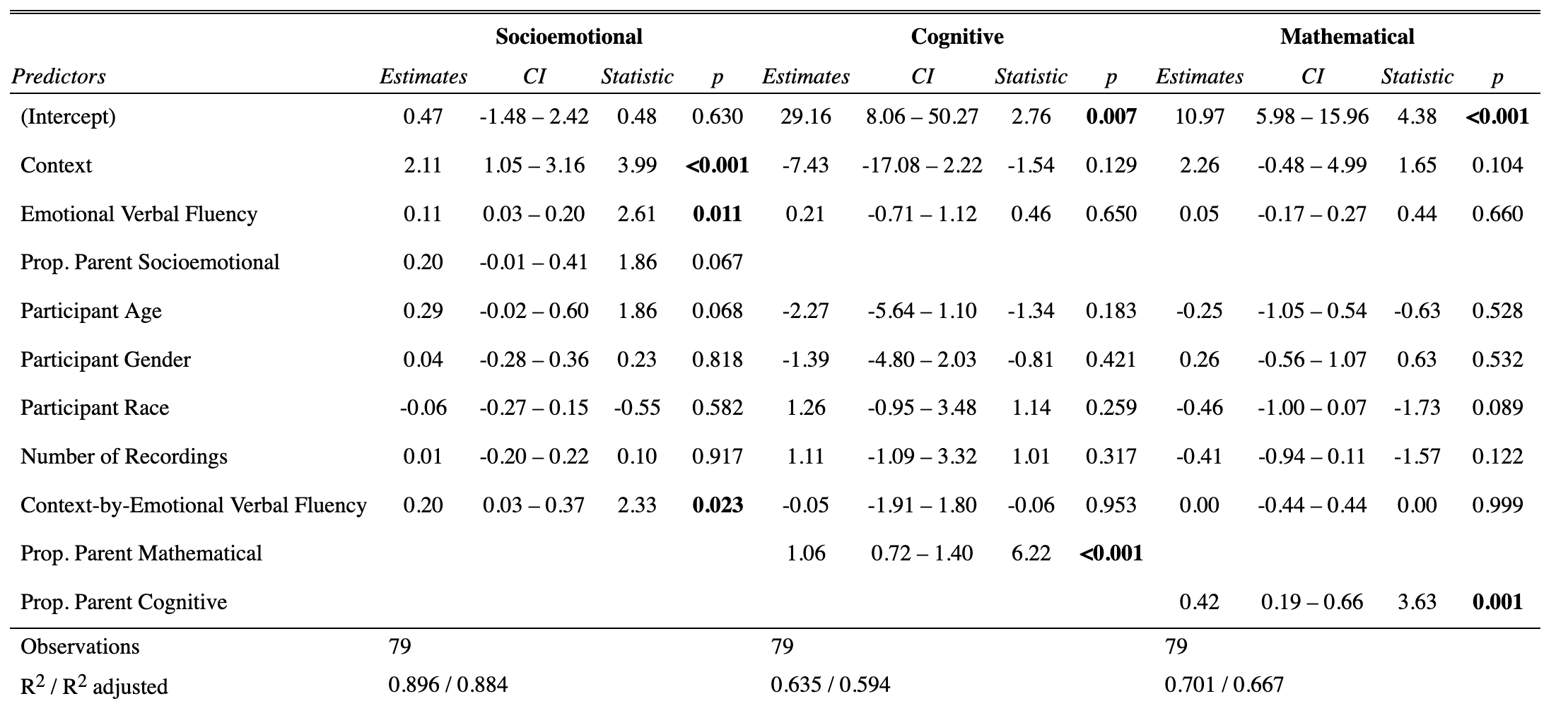


# Table S4. Full Model Output for Relationships Between Child and Parent Word Production (“Alignment”) Across Contexts

**
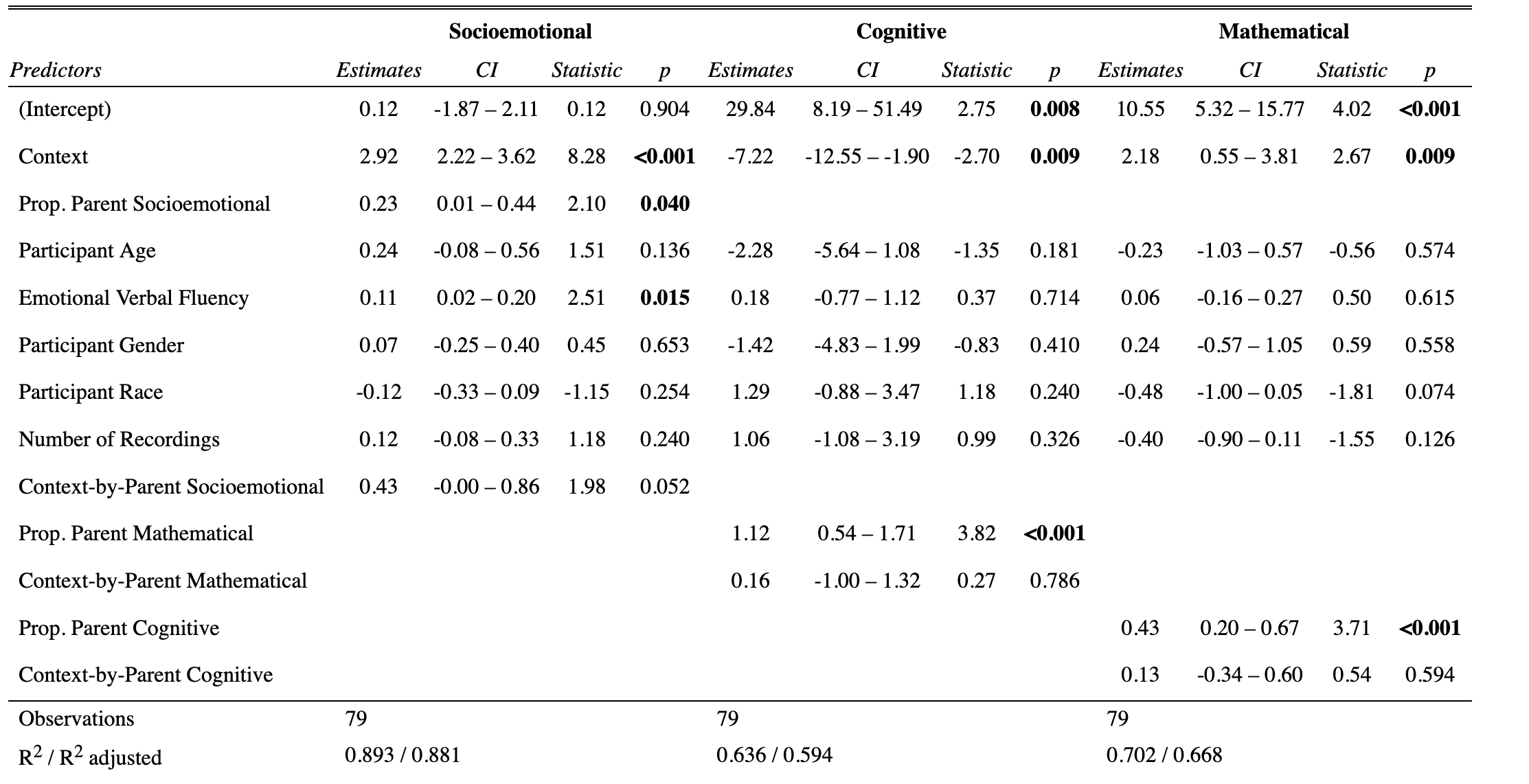
**

# Table S5. Full Model Output for Relationships between Conduct Problems, Callous-Unemotional Traits, and Child Word Production

**
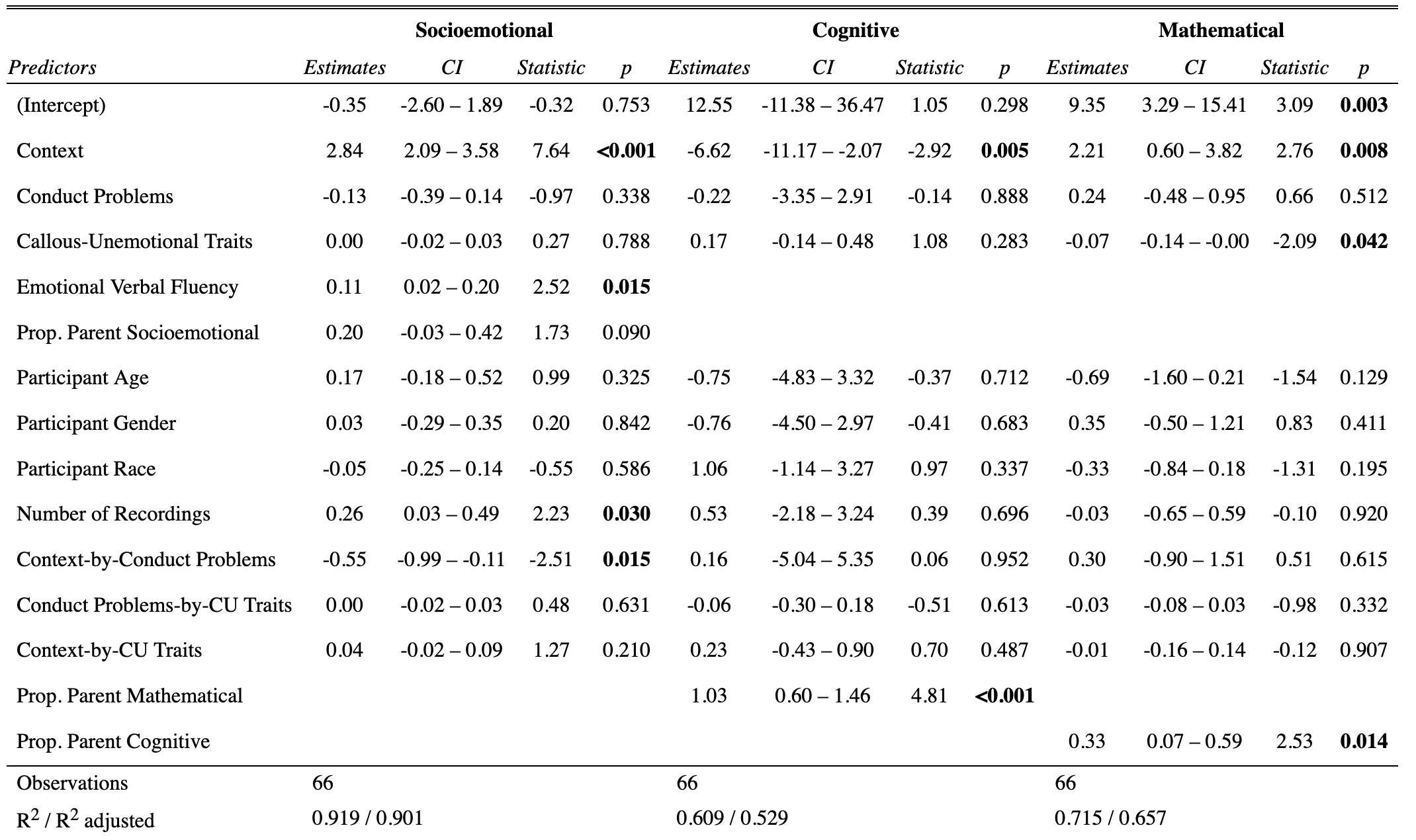
**

*Note.* In a preliminary model including the interaction between CP, CU traits, and context, the 3-way interaction was not significant.

#
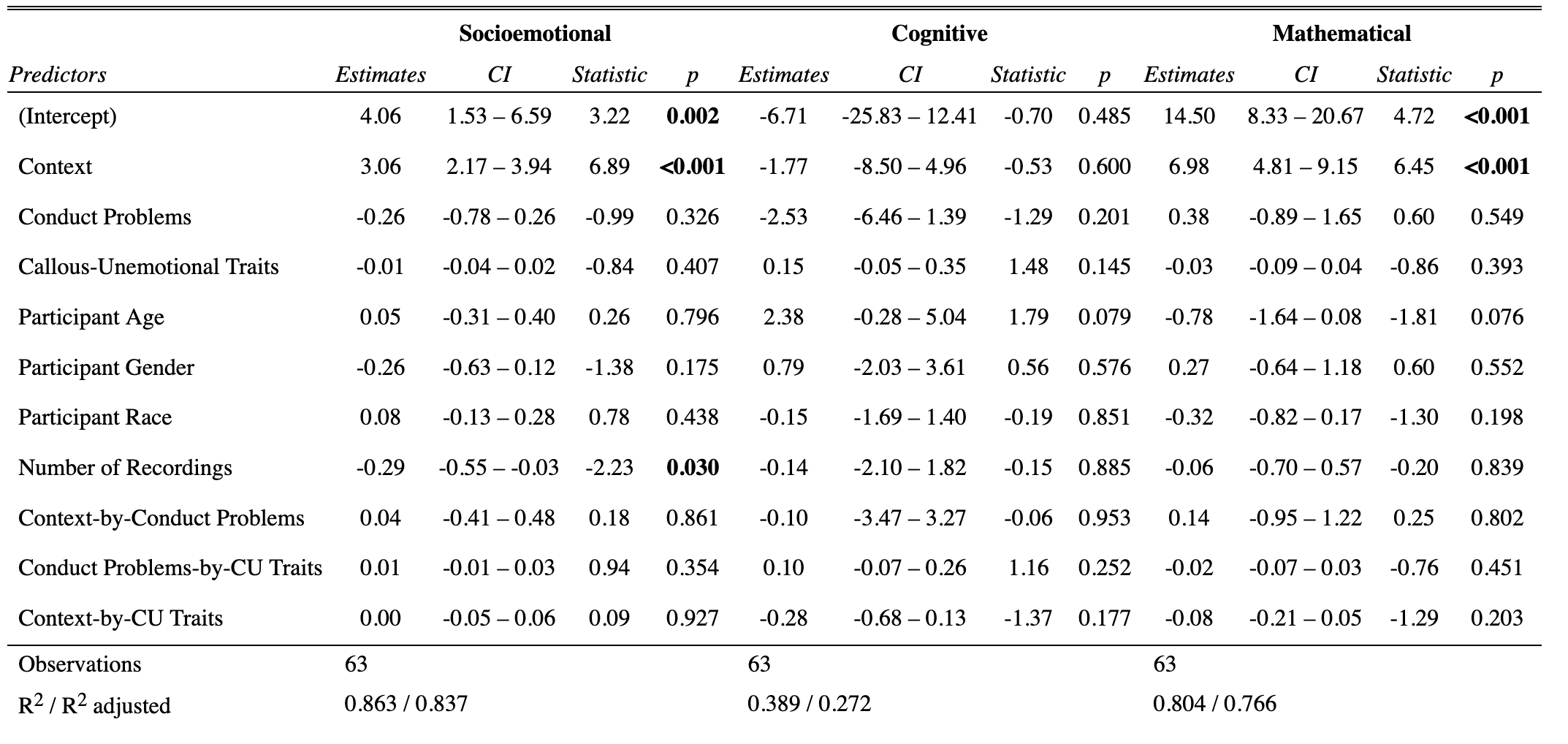
Table S6. Full Model Output for Relationships between Child Conduct Problems, Callous-Unemotional Traits, and Parent Word Production

# Table S7. Full Model Output for Relationships between Conduct Problems and Child and Parent Word Production (“Alignment”) Across Contexts

**
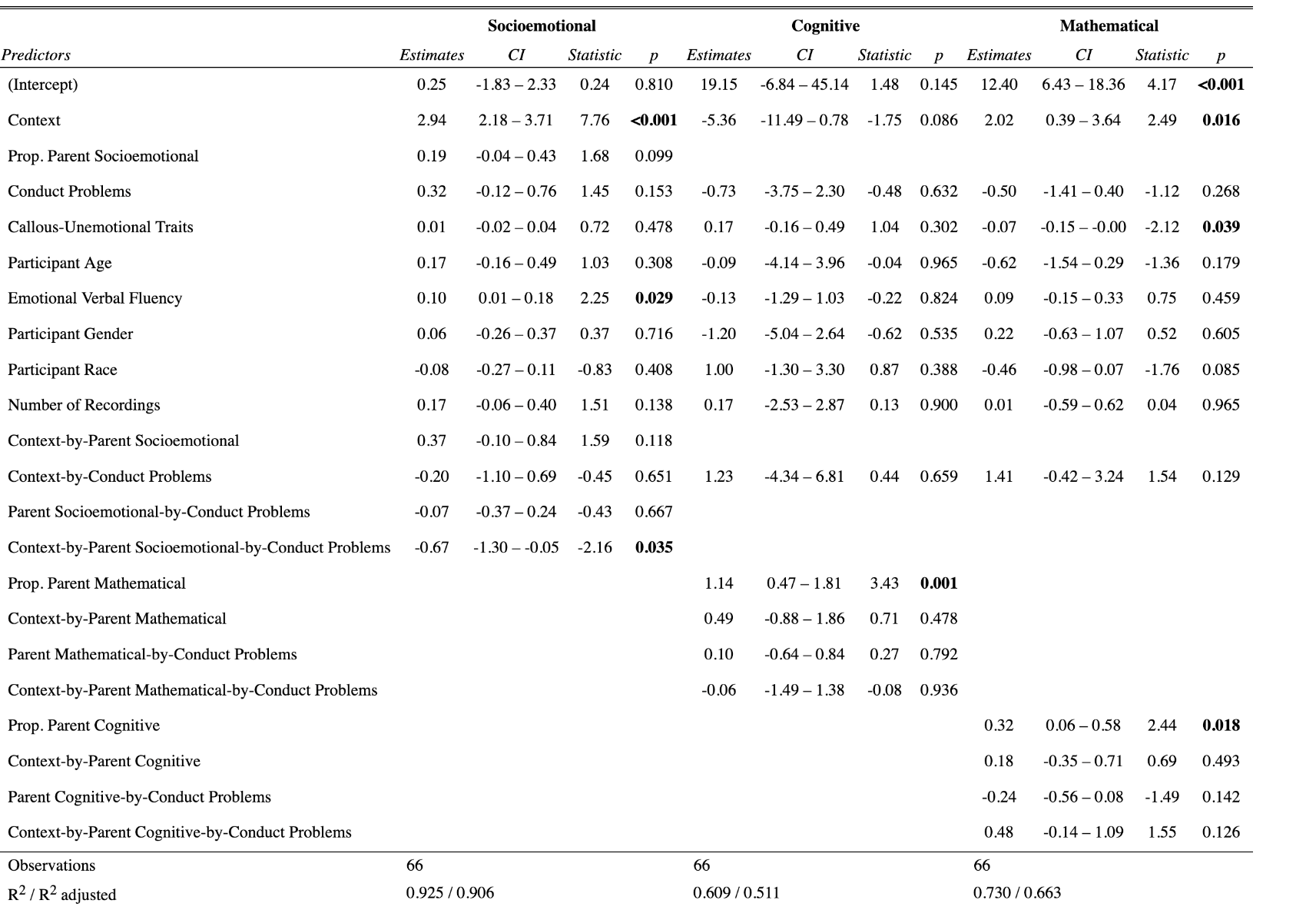
**

#
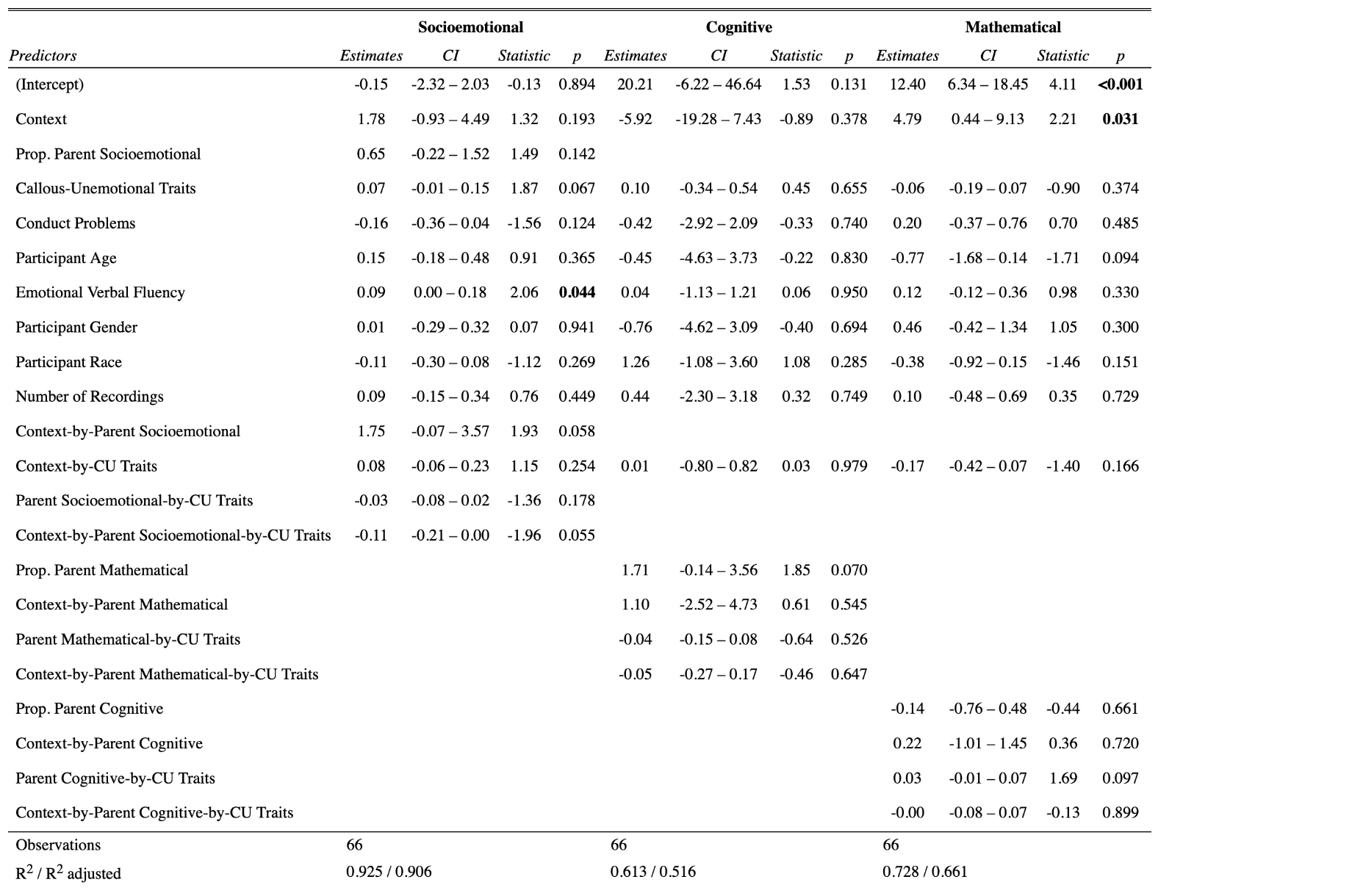
Table S8. Full Model Output for Relationships between Callous-Unemotional Traits and Child and Parent Word Production (“Alignment”) Across Contexts

# Table S9. Frequencies of the 14 Orders in which the Social Games were Played


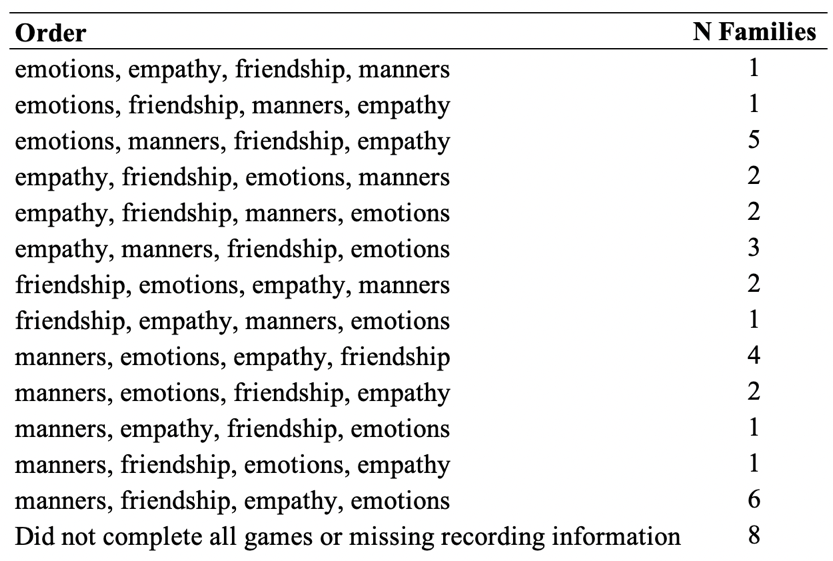


# Table S10. Mean Proportion of Number, Cognitive, and Socioemotional Words Produced in Individual Social Games

**
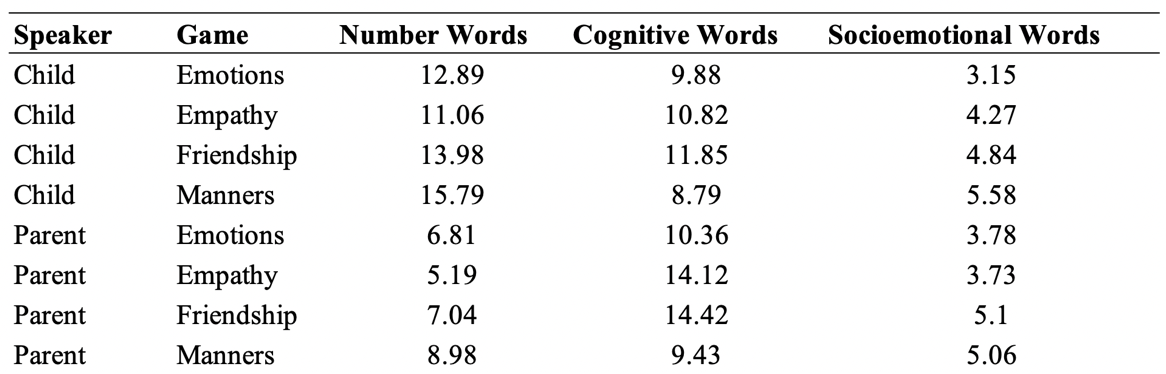
**

# Table S11. Social Board Games Pairwise Comparisons
